# Analysis of SARS-CoV-2 spike RBD binding to ACE2 and its inhibition by fungal cohaerin C using surface enhanced Raman spectroscopy

**DOI:** 10.1364/BOE.495685

**Published:** 2023-07-12

**Authors:** Christoph Wetzel, Linda Jansen-Olliges, Marc Stadler, Frank Surup, Carsten Zeilinger, Bernhard Roth

**Affiliations:** 1 Leibniz University Hannover, Hannover Centre for Optical Technologies, Nienburger Str. 17, 30167 Hannover, Germany; 2 Leibniz University Hannover, Centre of Biomolecular Drug Research, Schneiderberg 38, 30167 Hannover, Germany; 3 Helmholtz Centre for Infection Research GmbH, Department Microbial Drugs, Inhoffenstraße 7, 38124 Braunschweig, Germany; 4 Technische Universität Braunschweig, Institute of Microbiology, Spielmannstraße 7, 38106 Braunschweig, Germany; 5 Leibniz University Hannover, Cluster of Excellence PhoenixD, Welfenplatz 1, 30167 Hannover, Germany

## Abstract

The structure of the SARS-CoV-2 spike RBD and human ACE2 as well as changes in the structure due to binding activities were analysed using surface enhanced Raman spectroscopy. The inhibitor cohaerin C was applied to inhibit the binding between spike RBD and ACE2. Differences and changes in the Raman spectra were determined using deconvolution of the amide bands and principal component analysis. We thus demonstrate a fast and label-free analysis of the protein structures and the differentiation between bound and unbound states. The approach is suitable for sensing and screening and might be relevant to investigate other protein systems as well.

## 1. Introduction

The SARS-CoV-2 virus with its many variants is an obstacle to accurate and reliable detection as well as to appropriate sustainable immune defence strategies. The individual virus variants have different infectivity in the population, infiltrate the immunological defences and constantly trigger new waves of infection despite successful rapid vaccine development. This is due to a high permutation rate of certain virus areas that are also relevant for the immune defence [1–5]. A relevant entry into the cell is connected with the high affinity for the ACE2 receptor, which is found on the outer side of many cell types [1,5–7]. In the last years, existing detection methods in conjunction with vaccination strategies have become established around the world and have shown their effectiveness in limiting outbreaks in the population [1–5]. On the other side, this gives the virus and its subtypes a great deal of leeway to continuously cause new dangerous outbreaks. Cost-effective methods are needed to identify the characteristics and different types of the virus. Established methods for detection of the SARS-CoV-2 virus are for example real-time quantitative polymerase chain reaction (RT-qPCR) and enzyme-linked immunosorbent assays (ELISA) [8–10]. Biosensors coated with metal nanoparticles or graphene can enable an improvement as well as a fast and sensitive detection of the virus [10–12]. However, these techniques are good for detection but not suitable for structural analysis which is important to determine the different types of a virus and develop defence strategies. X-ray crystallography or cryogenic electron microscopy are able to investigate and visualize the molecule structure of the SARS-CoV-2 spike receptor binding domain (RBD) and the ACE2 receptor in detail, but this is relatively time and cost intensive [6,13,14]. Raman-based detection methods, in particular surface enhanced Raman spectroscopy (SERS), have several advantages as label-free, non-invasive and in situ optical methods and also provide fast results to detect and analyse the molecule structure of different proteins, bacteria and virus types including the SARS-CoV-2 virus. [9,15–24].

In previous work, a Raman signature of heat stress proteins (HSP90, HSP70) that can serve as a biomarker could be identified and it was also possible to identify temperature-dependent state changes of the human connexin channel Cx26 via resonance Raman spectroscopy [16]. Furthermore, the tetramer formation of bacterial potassium channel KcsA from Streptomyces lividans by evaluation of the change of the Raman signature could be determined [17]. This was possible because the protein amounts were sufficiently large, whereas this is often not the case with in situ detection scenarios such as investigating SARS-CoV-2. To measure also small amounts of SARS-CoV-2 and its proteins, there are several approaches to analyse and detect the virus with surface enhanced Raman spectroscopy [9,15,20–25]. Combined with machine learning, SERS is capable of detecting or classifying different types of the virus [9,25]. Also, customized and improved SERS substrates optimise the detection for small amounts of the virus [20,21,23].

In this work, we demonstrate a SERS-based structural analysis of the spike receptor binding domain (RBD) and the ACE2 receptor binding and its prevention by the inhibitor fungal cohaerin C. The inhibition of the spike RBD to ACE2 binding by cohaerin C was described in previous work by Jansen-Olliges et al. [26]. For this study, four different samples, which include the ACE2 receptor, the fragment of the spike protein as well as mixed samples with ACE2 and the spike RBD (AS-complex) and ACE2, the spike RBD and the inhibitor fungal cohaerin C (ASI-complex) were investigated. We analysed differences and changes in the structure of these four scenarios using deconvolution of the amide bands and principal component analysis (PCA) of the measured SERS spectra. The presented approach demonstrates that a rapid and label-free discrimination of the protein structure of the SARS-CoV-2 spike RBD and the ACE2 receptor as well as the observation of structural changes due to binding interactions is possible. This opens the potential for future sensing and screening applications in various other fields as well [27–29].

## 2. Materials and methods

### 2.1 Measurement principle

**Fig. 1.**
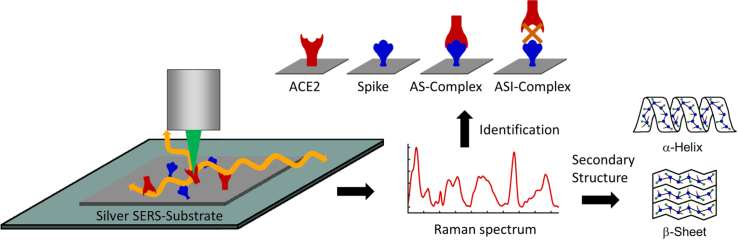
SERS measurement scheme. The proteins are prepared on a silver SERS-substrate and afterwards illuminated with laser light at a wavelength of 532 nm. The Raman scattered light is collected and the Raman spectrum is analysed. Differences and similarities are determined from four different samples: The ACE2 receptor, the spike RBD, a mixed sample with ACE2 and spike RBD (AS-complex) and a mixed sample with ACE2, spike RBD and the inhibitor (ASI-complex). With the help of the Raman spectrum the relative amount of secondary structures can be investigated and the four different scenarios can be identified.

The measurement method with the four different scenarios can be seen in Fig. 1. In the AS-complex insights into the changes of the structure of the bound ACE2 receptor and spike RBD can be investigated while in the ASI-complex this binding is prevented by the inhibitor cohaerin C. For a structural analysis, the deconvolution of the amide bands and the principal component analysis (PCA) reveal information about the primary and the secondary structure in form of α -helical and β -sheet structures. The distribution and changes of Raman signals associated with the secondary structure can be used to differentiate between the samples. Consequently, a determination of bound and unbound states of the spike RBD and the ACE2 receptor is possible.

### 2.2 Materials

A fragment of the spike protein with ACE2 binding activity was purchased from Genaxxon bioscience (S5340.0100) corresponding to accession no. YP_009724390.1 containing the sequence: RVQPTESIVRFPNITNLCPFGEVFNATRFASVYAWNRKRISNCVADYSVLYNSASFSTFKCYGVSPTKLNDLCFTNVYADSFVIRGDEVRQIAPGQTGKIADYNYKLPDDFTGCV IAWNSNNLDSKVGGNYNYLYRLFRKSNLKPFERDISTEIYQAGSTPCNGVEGFNCYFP LQSYGFQPTNGVGYQPYRVVVLSFELLHAPATVCGPKKSTNLVKNKCVNF. Cohaerin C was isolated in a similar manner as described previously [26,30] from stromata of Jackrogersella cohaerens, collected from Fagus sylvatica wood. The binding affinity of cohaerin C was described and characterized by Jansen-Olliges et al. [26].

Silver coated SERS substrates from Ato ID (Vilnius, Lithuania) were used. According to the product specification the substrate is made of an stochastic nano-pattern with a size from nano- to micrometers. Analytes on the substrate can experience a hot spot, which can lead to a strong enhancement of the Raman scattering. To compensate this phenomenon a wide laser spot of at least 20 μ m diameter is needed to average signals from hot spots and surrounding nano-patterns. The substrate is manufactured to enhance most of the Raman signal with an relative enhancement ratio of $10^{6}$ . Regarding reproducible measurements a deviation of 10% is very common.

### 2.3 Expression and purification of ACE2

The DNA encoding the human ACE2 receptor was codon usage optimized, synthesized, and provided in a pET28a bacterial expression vector (by Synbio Technologies LLC, Monmouth Junction, NJ, USA) named pET28a_hACE2. The plasmid encoding human ACE2 was transfected into E. coli BL21(DE3) cells and pre-cultures were prepared. The next day, the main cultures (Terrific Broth) were inoculated with precultures (1:50) and incubated at 37 °C for 6 h. The cell suspension was cooled to 8 °C, induced with 1 mM isopropylthiogalactoside (IPTG), and maintained at 8 °C for 70 h. After harvesting by centrifugation (8,500 xg, 15 min) the cells were either stored at -80 °C or directly resuspended in buffer A (20 mM Tris-HCl, pH 8.0, 50 mM KCl, 2 mM β -mercaptoethanol, 2 mM imidazole, 0,05 % Tween-20, 10 % glycerol) and lysed twice with a French press using 14,000-16,000 p.s.i.. Cell lysates were incubated with 2.5 % glycerol, 2.5 % PEG400, 1 % N-lauroylsarcosin, 5 M urea for 1 h at 37 °C and subsequently centrifuged at 20,000 xg and 4 °C for 1 h. A Ni-Indigo column (Cube Biotech) was equilibrated with buffer A (20 mM Tris-HCl pH 8.0, 50 mM KCl, 0,05 % Tween-20, 2 mM β -mercaptoethanol, 10 % glycerol) and supernatant (1:2 diluted with lysis buffer) was loaded on the column. Hexa-histidin-tagged (6x-His) human ACE2 was eluted with 500 mM imidazole in buffer A and further purified by size-exclusion chromatography using buffer A and concentrated to 3 mg/mL using Amicon centrifugal spin filters (30K) in storage buffer (20 mM Tris-HCl, pH 7.5, 50 mM KCl, 2 mM β -mercaptoethanol, 10 % (v/v) glycerol). The presence and purity of the protein were detected by SDS-PAGE and immune blotting using monoclonal anti-His antibody (antibodies-online GmbH, Germany) and secondary anti-mouse alkaline phosphatase (Merck KGaA, Germany). The susceptibility assay of the of recombinant purified ACE2 receptor for spike and substances was described earlier [26].

### 2.4 Sample preparation

The ACE2 receptor and the spike RBD were prepared on silver coated SERS substrates RandaS (Ato ID, Vilnius, Lithuania). After dipping the substrate for 10 s in 20 % ethanol and following drying, 9 μ M of ACE2 and the spike protein fragment were applied in PBS buffer on the SERS substrate. The proteins were incubated for 16 h at 4 °C in a wet chamber and then dried. Afterwards Raman spectra of the proteins were measured with an in-house Raman spectroscopy setup. Investigated samples were the ACE2 receptor, the SARS-CoV-2 spike RBD, ACE2 mixed with the spike RBD and ACE2 mixed with the spike RBD and the inhibitor fungal cohaerin C. Mixed samples were prepared with 9 μ M of each protein and 1 % of dimethyl sulfoxide (DMSO) in PBS buffer. The sample with the inhibitor fungal cohaerin C was prepared in the same way with additional 150 μ M of the inhibitor.

### 2.5 Raman setup and spectra processing

**Fig. 2.**
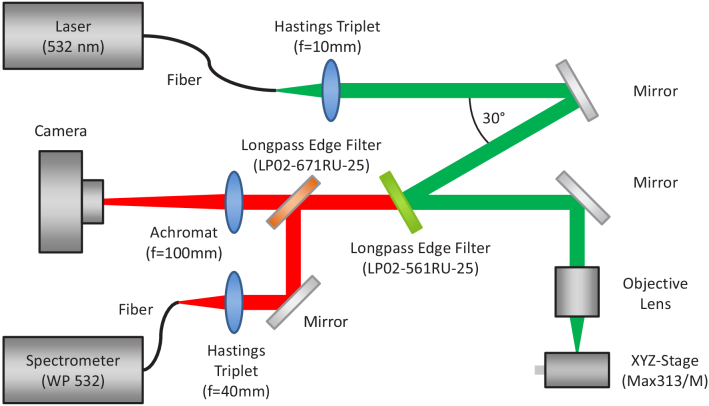
Schematic measurement setup. The laser light is collimated by a hastings triplet, reflected by the longpass edge filter and focused on the sample by an objective lens. Raman scattered light is collected and collimated with the same objective lens and focused into the collection fiber by a second hastings triplet. Spectra are recorded by the spectrometer. The prepared sample was placed on a xyz-stage.

Surface enhanced Raman measurements were done with an in-house Raman spectroscopy setup with an excitation wavelength of 532 nm which can be seen in Fig. 2. For excitation the fiber coupled 532 nm solid-state laser RLTMLL-532-200 from Roithner Lasertechnik GmbH (Wien, Austria) was used. First, the laser light of the fiber is collimated by the hastings triplet TRH064-010-A from Thorlabs GmbH (Munich, Germany) and reflected in a 30° angle by the longpass edge filter LP02-561RU-25 (Semrock inc., USA). Then the laser light is focused for excitation of the sample by the objective lens NPL Fluotar 100x from Leitz Wetzlar. Raman scattered light is then collected and collimated by the same objective lens, reflected by the longpass edge filter LP02-671RU-25 (Semrock inc., USA) and focused by the hastings triplet TRH254-040-A into the fiber bundle BFL105LS02 from Thorlabs GmbH (Munich, Germany). The collection fiber is connected to the Raman spectrometer WP 532 (Wasatch Photonics inc., USA) where the Raman scattered light is analysed. The position of the prepared samples was adjusted using the xyz-stage Max313D/M from Thorlabs GmbH. To control if the sample is visually damaged the camera Altair GPCAM2 AR0130 Mono (Altair Astro, United Kingdom) was used. The setup is similar to the one described in Kniggendorf et al. [31].

For the measurements, Raman spectra of different positions on the SERS substrate were taken with an integration time of 10 s. To take the variations of the measured spectra into account, at least 70 different positions on each sample were measured. The spot size of the laser and the detection area size on the sample was about 450 μ $m^{2}$ . The maximum laser power on the samples during the measurements was 5 mW. To analyse and compare the results, the measured Raman spectra were processed in Matlab (The MathWorks, Inc.,USA) in the following way: First the dark spectrum was subtracted and the spectra were smoothed with the locally estimated scatterplot smoothing (loess) algorithm using the in Matlab implemented function smoothdata. Because the PBS buffer caused a fluorescence signal, the SERS spectrum was baseline corrected by the algorithm of Matthias Koch et al. [32]. Here, a bandwidth of 200 ${\mathrm{cm}}^{-1}$ was applied in the algorithm, being the maximal width of the Raman bands in the investigated region. In a last step, for better comparison, the Raman spectra were vector normalised for the region 1200 ${\mathrm{cm}}^{-1}$ to 1750 ${\mathrm{cm}}^{-1}$ , as the background glass signal from the SERS substrate can influence the region 800 ${\mathrm{cm}}^{-1}$ to 1200 ${\mathrm{cm}}^{-1}$ . The Raman spectra processed in this way were analysed using deconvolution of the Raman bands and principal component analysis (PCA).

For the deconvolution process of the amide bands the positions of hidden peaks in these bands can be determined by using the second derivative of the Raman spectra [33,34]. To obtain more consistent results, the positions of the peaks were calculated in a Raman spectrum which was averaged over all measurements of each sample. Afterwards, the determined positions were used as starting parameters to fit signals with a Gaussian profile in Raman spectra which were averaged over 5 spectra. Using the function fit in Matlab with the Trust-Region algorithm the signals in the amide band were computed. The minimal bandwidth for fitted signals was 9 ${\mathrm{cm}}^{-1}$ being the minimal spectral resolution of the optical setup. Since the amide bands were normalised for the analysis, the amplitude can vary from 0 to 1. The area of the fitted Gaussian profiles was used to determine the relative amount of secondary structure components within the amide bands. Standard deviations were calculated from the determined hidden peaks of multiple Raman spectra for different measurement positions. The measurement and spectra processing of one measurement spot takes approximately 20 s on average.

The principal component analysis was performed with the function pca in Matlab. Here, the spectra were not averaged as it was the case in the deconvolution process.

## 3. Results

**Fig. 3.**
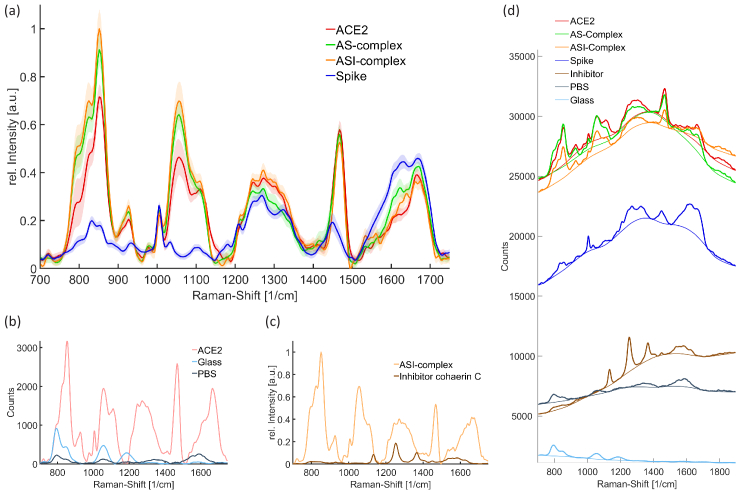
SERS spectra of the samples and the background. The spectra in (a-c) are baseline corrected. (a) The mean Raman spectra of ACE2, spike RBD, the ACE2-spike complex (AS-complex) and the ACE2-spike-inhibitor complex (ASI-complex) are shown. The Raman spectra are baseline corrected and for a better comparison vector normalised for the region 1200 ${\mathrm{cm}}^{-1}$ to 1750 ${\mathrm{cm}}^{-1}$ . The shaded area around the graphs shows the standard deviation of the measurements. (b) Possible background signals of PBS and the glass substrate. If the glass signal is stronger the PBS signal is weaker and vice versa. (c) Signals of the ASI-complex and fungal cohaerin C in PBS. (d) SERS spectra with fluorescence signal and the computed baseline.

SERS spectra of the ACE2 receptor and the SARS-CoV-2 spike RBD were obtained and analysed for the four different scenarios described above. In Fig. 3 the mean Raman spectra of all measured positions on the SERS substrate are shown before and after baseline correction. Additionally, possible background signals are presented. Assigned signals of the spectra are described in Table 1. The signal positions in the Raman spectra are similar and can be assigned to same bonds. The analysis is split in the region from 800 ${\mathrm{cm}}^{-1}$ to 1200 ${\mathrm{cm}}^{-1}$ and the region of amide bands between 1200 ${\mathrm{cm}}^{-1}$ and 1750 ${\mathrm{cm}}^{-1}$ .

**Table 1.** Signals of the measured SERS spectra from ACE2 (A), spike RBD (S), the ACE2-spike complex (AS) and the ACE2-spike-inhibitor complex (ASI).

Raman-Shift	Sample	Assignments
827 ${\mathrm{cm}}^{-1}$	All	Tyrosine [35–39]
854 ${\mathrm{cm}}^{-1}$	All	Tyrosine [35–39]
899 ${\mathrm{cm}}^{-1}$	All	Tryptophan [36,37]
927 ${\mathrm{cm}}^{-1}$	A,AS,ASI	α -helix, N-C-C, C-C [36,37,39]
934 ${\mathrm{cm}}^{-1}$	S	α -helix, N-C-C, C-C [36,37,39]
978 ${\mathrm{cm}}^{-1}$	A,AS,ASI	Tryptophan [37]
1005 ${\mathrm{cm}}^{-1}$	All	Phenylalanine [36,37]
1030 ${\mathrm{cm}}^{-1}$	All	Phenylalanine [36,37]
1054 ${\mathrm{cm}}^{-1}$	A,AS,ASI	C-O, C-C [36]
1090 ${\mathrm{cm}}^{-1}$	All	C-N, C-C [35,36]
1110 - 1130 ${\mathrm{cm}}^{-1}$	A,AS,ASI	C-N, C-C [35–37]
1170 - 1185 ${\mathrm{cm}}^{-1}$	S,AS,ASI	Tyrosine, Phenylalanine [39]
1205 - 1215 ${\mathrm{cm}}^{-1}$	All	Phenylalanine, Tyrosine [35,36,38]
1220 - 1240 ${\mathrm{cm}}^{-1}$	All	β -sheet [35,36,38,40]
1240 - 1270 ${\mathrm{cm}}^{-1}$	All	unordered structure [35,36,38,40]
1265 - 1280 ${\mathrm{cm}}^{-1}$	All	α -helix [36,38]
1281 - 1300 ${\mathrm{cm}}^{-1}$	All	α -helix [35,36,38,40]
1320 ${\mathrm{cm}}^{-1}$	All	C-H (def) [35–37]
1340 ${\mathrm{cm}}^{-1}$	All	Tryptophan [36]
1356 ${\mathrm{cm}}^{-1}$	All	Tryptophan [36]
1440 ${\mathrm{cm}}^{-1}$	S,AS	C-H (def) [36,37]
1465 ${\mathrm{cm}}^{-1}$	A,AS,ASI	C-H (def) [36,37]
1560 ${\mathrm{cm}}^{-1}$	All	Tryptophan [37]
1580 ${\mathrm{cm}}^{-1}$	All	Phenylalanine, Tyrosine [39]
1605 ${\mathrm{cm}}^{-1}$	All	Phenylalanine [35,37,38]
1616 ${\mathrm{cm}}^{-1}$	All	Tyrosin, Tryptophan [35,37,38]
1620 - 1645 ${\mathrm{cm}}^{-1}$	All	β -sheet [33,35,36,41]
1645 - 1665 ${\mathrm{cm}}^{-1}$	All	α -helix [33,35,36,38,39,41]
1665 - 1680 ${\mathrm{cm}}^{-1}$	All	β -sheet [33,35,36,41]
1660 - 1700 ${\mathrm{cm}}^{-1}$	All	unordered structure/ β -turn [33,35,36,41]

### 3.1 Analysis of the spectral region 800 ${\mathrm{cm}}^{-1}$ to 1200 ${\mathrm{cm}}^{-1}$

From the spectra shown in Fig. 3(a), it can be seen, that in the region from 800 ${\mathrm{cm}}^{-1}$ to 1200 ${\mathrm{cm}}^{-1}$ the shape and intensity of the ACE2 sample and both mixed samples are very similar in comparison to the measured spectrum of the spike RBD. All spectra show signals at 827 ${\mathrm{cm}}^{-1}$ and 854 ${\mathrm{cm}}^{-1}$ which can be assigned to the tyrosine doublet [35–39]. It needs to be considered that in the presented spectra the signals of the glass background around 800 ${\mathrm{cm}}^{-1}$ and 854 ${\mathrm{cm}}^{-1}$ are included. Another feature of the SERS spectrum of ACE2 is a more intense peak at 927 ${\mathrm{cm}}^{-1}$ , which originates from protein N-C-C bonds and α -helical structures [36,37,39]. In the spectrum of the spike RBD this signal is shifted to 934 ${\mathrm{cm}}^{-1}$ and is less intense. Peaks of phenylalanine at 1005 ${\mathrm{cm}}^{-1}$ and 1030 ${\mathrm{cm}}^{-1}$ can be detected in all samples [36–39]. Signals between 1050 ${\mathrm{cm}}^{-1}$ and 1180 ${\mathrm{cm}}^{-1}$ are caused by carbon bonds, e.g. C-H, C-N and C-O bonds [35–38]. In this range the Raman band in the SERS spectrum of the spike RBD is weaker than in the other SERS spectra. Here, as well, the glass background with a broad signal around 1054 ${\mathrm{cm}}^{-1}$ influences this part of the spectrum.

It can be concluded that in the region from 800 ${\mathrm{cm}}^{-1}$ to 1200 ${\mathrm{cm}}^{-1}$ in mixed samples signals of the ACE2 receptor are more dominant than the ones of the spike RBD. The mixed samples show similar features compared to the SERS spectra of ACE2. Differences caused by binding interactions are very weak in this section. Also it needs to be considered that the glass signal of the SERS substrate can influence results in this region, since the strength of the glass signals can vary.

A better comparison is possible in the region 1200 ${\mathrm{cm}}^{-1}$ to 1750 ${\mathrm{cm}}^{-1}$ since the glass substrate exhibits no signals there and the signal strength of the spike RBD and the ACE2 receptor are similar. This can be seen in Fig. 3(a) where the mean spectra of all measurements are shown. Differences caused by binding interactions can be identified more clearly. Also, the amide I band and amide III band of the Raman spectra, which display signals in this region, reveal information about the secondary structure of the proteins.

### 3.2 Analysis of the spectral region 1200 ${\mathrm{cm}}^{-1}$ to 1750 ${\mathrm{cm}}^{-1}$

In the following, the SERS spectra of the measured samples are analysed in the region 1200 ${\mathrm{cm}}^{-1}$ to 1750 ${\mathrm{cm}}^{-1}$ . The amide bands are deconvolved and differences and changes in the secondary structure are determined. Since signals in the amide bands can be enhanced slightly differently, because of the SERS effect or influences from the fluorescence, an exact determination of the distribution of the secondary structures is challenging. However, a determination whether a certain structure exists in larger quantity than others is possible.

#### 3.2.1 Comparison and changes of the secondary structure

**Fig. 4.**
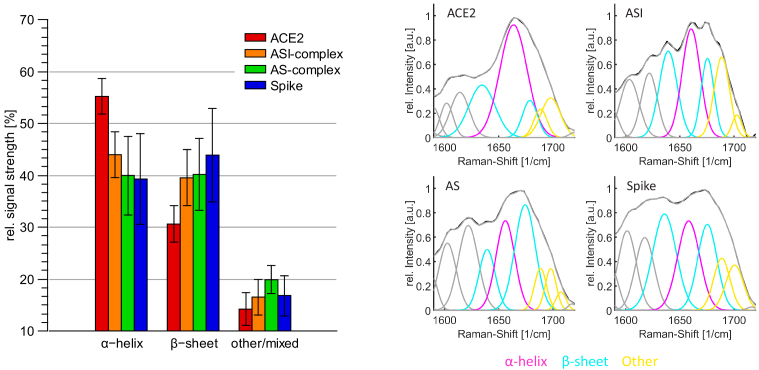
Left: The distribution of the secondary structure in the amide I band. The relative amount of the signal stemming from the β -sheet was determined by taking the average of the two signals around 1635 ${\mathrm{cm}}^{-1}$ and 1675 ${\mathrm{cm}}^{-1}$ . Random and other structures were averaged from signals between 1680 ${\mathrm{cm}}^{-1}$ and 1705 ${\mathrm{cm}}^{-1}$ . Right: Example of the deconvolved amide I band of the investigated samples. Raman spectra were measured and processed as described in the section 2.5.

To investigate the structural changes, first the amide I band is analysed. In Fig. 4 the deconvolved amide I band of the different samples, which were investigated, is shown. Here, signals around 1635 ${\mathrm{cm}}^{-1}$ and 1675 ${\mathrm{cm}}^{-1}$ can be assigned to a β -sheet structure [33,35,36,41]. The signal of the α -helical structure can be located in the range from 1645 ${\mathrm{cm}}^{-1}$ to 1665 ${\mathrm{cm}}^{-1}$ [33,35,36,38,39,41] and between 1660 ${\mathrm{cm}}^{-1}$ to 1700 ${\mathrm{cm}}^{-1}$ signals of unordered structures and β -turns can appear [33,35,36,41]. Hidden peaks within the amide I band, which explain the shape of the band, are found as described in section 2.5.

It can be seen that the deconvolved signal in the spectrum of ACE2 around 1661 ${\mathrm{cm}}^{-1}$ , which can be assigned to the α -helix, is very intense in comparison to the other signals in the amide I band. In particular, signals at 1637 ${\mathrm{cm}}^{-1}$ and 1676 ${\mathrm{cm}}^{-1}$ from β -sheets are less intense. In case of the spike RBD the intensity of signals of β -sheets are stronger than the signal at 1658 ${\mathrm{cm}}^{-1}$ originating from the α -helix. Thus, it can be concluded that ACE2 has a relatively larger quantity of α -helical structures while the spike RBD shows more characteristics of β -sheet structures.

The determined distribution and changes in the secondary structure of the spectra of the mixed samples can be seen in Fig. 4 on the left side. Compared to the ACE2 receptor, both samples have less intense alpha-helix signals and stronger peaks in the range of β -sheet structures. While the secondary structure of the AS-complex is more similar to the one of the spike protein fragment, the ASI-complex shows more characteristics of the ACE2 receptor. In the latter case the signal strength of α -helical structures is less in the AS-complex where the spike RBD binds to the ACE2 receptor. The Raman spectra of the ASI-complex, where the binding interaction of the spike RBD and ACE2 is inhibited, contain more intense signals of α -helical structures.

**Fig. 5.**
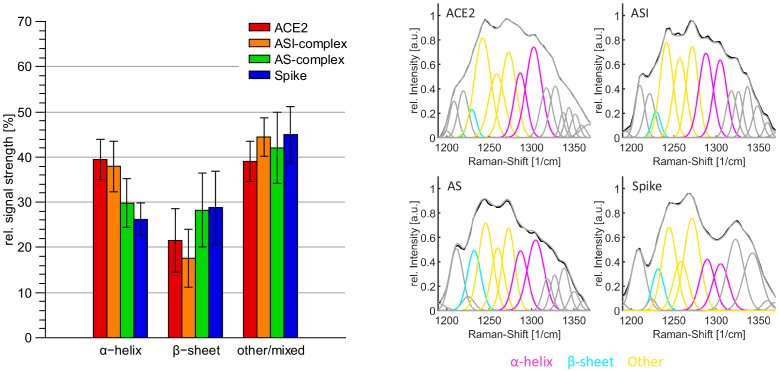
Left: The distribution of the secondary structure in the amide III band. Here, the relative amount of the α -helix was determined by taking the average of the two signals around 1280 ${\mathrm{cm}}^{-1}$ and 1300 ${\mathrm{cm}}^{-1}$ . Random and other structures were averaged from signals between 1240 ${\mathrm{cm}}^{-1}$ and 1270 ${\mathrm{cm}}^{-1}$ . Right: Example of the deconvolved amide III band of the investigated samples. Spectra are measured and processed as described in the section 2.5.

The amide III band can be additionally used to verify results of the amide I band analysis. The results in this amide band are less pronounced, because other bonds, such as C-H deformations, can also appear in this region and overlap or influence the signals. Consequently more mixed or other signals are expected. In Fig. 5 the deconvolved amide III bands for the four different types of samples are shown. In this band, signals from 1220 ${\mathrm{cm}}^{-1}$ to 1240 ${\mathrm{cm}}^{-1}$ can be assigned to a β -sheet structure [35,36,38,40], whereas peaks of unordered structures can be found in the range of 1240 ${\mathrm{cm}}^{-1}$ to 1270 ${\mathrm{cm}}^{-1}$ [35,36,38,40]. Signals of the α -helical structure are between 1265 ${\mathrm{cm}}^{-1}$ and 1300 ${\mathrm{cm}}^{-1}$ [35,36,38,40].

From the deconvolution in Fig. 5 it can be seen, that the differences in the distribution of the secondary structure are not as clear as it is the case in the amide I band. Peaks around 1245 ${\mathrm{cm}}^{-1}$ and 1270 ${\mathrm{cm}}^{-1}$ were considered as mixed signals since here also signals of unordered or random coil structures can influence this area. However, the ratio of Raman signals from β -sheet and α -helical structures is similar to the results of the amide I band. The ACE2 sample and the ASI-complex have more intense signals of the α -helical structure while in the spectra of the spike RBD and the AS-complex the α -helical structure is less intense and the peak in the range of the β -sheet structure is relatively strong in comparison to the other two types of samples.

**Fig. 6.**
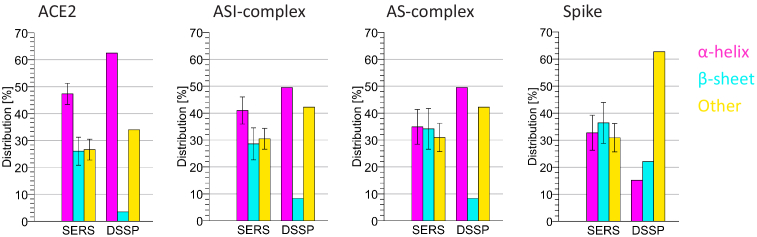
Comparison of data from the SERS analysis with X-ray diffraction data of human ACE2 and chimeric SARS-CoV-2 RBD from the protein data base (PDB) according to PDB ID 6vw1. Determined secondary structures from SERS measurements of the amide I and amide III bands are combined in this comparison.

For a comparison with the secondary structure of X-ray diffraction analysis from data of the protein data bank (PDB) according to PDB ID 6vw1 [42,43], the Define Secondary Structure Proteins (DSSP) algorithm [44] was applied using the secondary structure server [45]. ACE2 and spike RBD measurements were compared to the structure of the human ACE2 and the chimeric SARS-CoV-2 RBD, whereas the mixed samples were compared to the structure of the whole complex according PDB ID 6vw1 [42,43]. The comparison with the X-ray diffraction data shows that the SERS estimation of the relative amount of the α -helix and β -sheet structures of individual samples, such as ACE2 and the spike RBD, is consistent with this method. In Fig. 6 it can be seen that the ratio between α -helix and β -sheet structures is similar. In case of the ACE2 sample, most differences can be observed in the β -sheet structure. Since overlapping of the signals in the amide bands is still possible, a larger variance between the SERS estimation and the X-ray diffraction data of few existing structures, such as the β -sheet structure, can be expected. Other structures than α -helix and β -sheet cannot be compared completely since not all structures can be determined in the amide bands of the Raman spectra very well. Consequently, larger differences of other structures between the two analysis methods can appear. In case of the mixed samples the ASI-complex also shows similar distributions compared to the X-ray diffraction data of the whole complex. Here, the determined structure from the SERS spectra is still dominated by Raman signals of α -helical structures, but in comparison to ACE2 also the amount of β -sheet structures from the spike RBD increased. This change can be seen very well in both mixed samples. However, the AS-complex differs more from the X-ray diffraction data. Here the sheet structures in the SERS analysis are more intense and more similar to the secondary structure of the spike RBD.

#### 3.2.2 Differentiation of measured SERS-spectra using principal component analysis

**Fig. 7.**
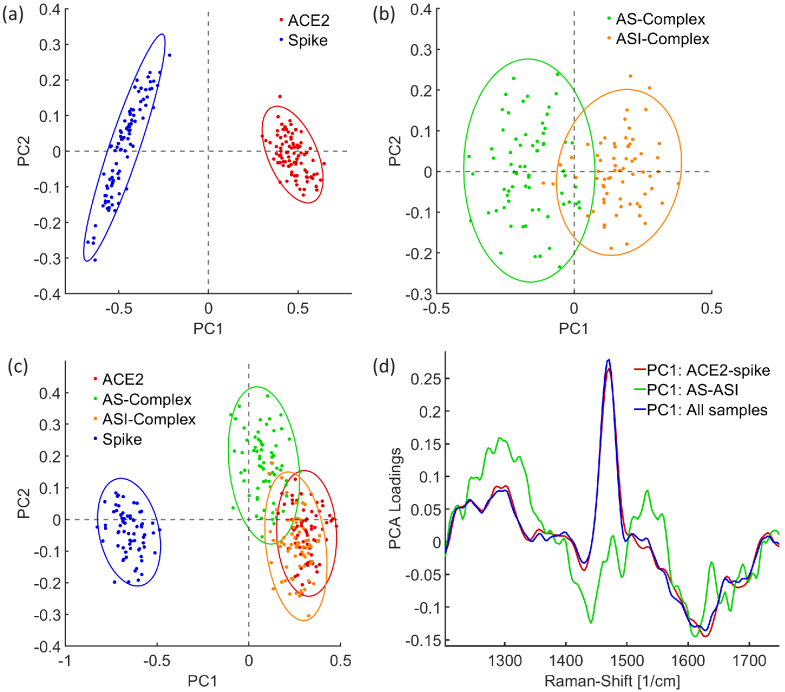
PCA analysis of ACE2, spike RBD, the AS-complex and the ASI-complex. Each point in the scatter plots describes a measurement on a different position on the SERS substrates (a-c). Raman spectra were measured and prepared as described in section 2.5. (a) Score plot of each measurement from ACE2 and spike. The Raman spectra can be separated by the 1st principal component. (b) Score plot of each measurement from the AS-complex and the ASI-complex. Also, here the spectra can be distinguished by the 1st principal component. (c) Score plot from the PCA of each measurement from all samples. While the ASI-complex shows nearly the same features as ACE2, the measurements of the AS-complex are shifted towards the score from the spike RBD. (d) The 1st principal components of the comparisons in (a), (b) and (c).

With the principal component analysis (PCA) the features of each SERS spectrum can be determined and a distinction between individual measurements is possible. In comparison to the analysis of the deconvolved spectra, the ratios and strength of the different Raman bands are considered. In the PCA the same amount of measurements for each sample was used, so that the influence on the analysis is the same. Here, the spectra are compared and analysed in the spectral region between 1200 ${\mathrm{cm}}^{-1}$ and 1750 ${\mathrm{cm}}^{-1}$ , because, as explained earlier, at lower wavenumbers Raman signals of ACE2 are dominant, differences are less pronounced and the background signal of the glass substrate disturbs the measurements. In Fig. 7 the results of the PCA are shown. The PCA of the spike RBD and the ACE2 spectra displays that these two samples are well distinguishable and each Raman spectrum has certain characteristics (Fig. 7 (a)). The measurements can be separated by the first principal component (PC) which explains 88.36 % of the variance. The other principal components explain variations which exist in all spectra in the same way and consequently cannot be used for determining differences. By comparing ACE2 and the spike protein fragment, the normalised Raman spectra of ACE2 show a relative strong amide III band and an intense peak around 1465 ${\mathrm{cm}}^{-1}$ . The normalised spectra of the spike protein have a peak around 1440 ${\mathrm{cm}}^{-1}$ and the intensity of the amide I band is higher than in the Raman spectra of the ACE2 receptor. In fact, the signal responsible for C-H deformations is slightly shifted and the ratio of the amide I and amide III band is different.

Regarding differences in the shape of the amide bands, the ACE2 spectra have more intense peaks at 1245 ${\mathrm{cm}}^{-1}$ and 1290 ${\mathrm{cm}}^{-1}$ whereas the whole region from 1270 ${\mathrm{cm}}^{-1}$ to 1310 ${\mathrm{cm}}^{-1}$ is more dominant as shown in the first principal component (Fig. 7 (d)). From that, it can also be concluded that the quantity of the α -helical structure is larger in the ACE2 receptor. Also, as the whole amide III band is more intense, a determination of the spike RBD features is more challenging. In the amide I band the Raman signal of the spike protein fragment is relatively stronger than in the spectrum of the ACE2 receptor. Here, in relation, the first principal component shows maxima around 1628 ${\mathrm{cm}}^{-1}$ and between 1670 and 1685 ${\mathrm{cm}}^{-1}$ which can be assigned to the features of the β -sheet and β -turn structures. Consequently, the Raman spectra of the spike RBD reveal more characteristics of a β -sheet structure.

The PCA of the AS-complex and the ASI-complex (Fig. 7 (b)) shows that both types of samples can be separated by the first principal component. While the AS-complex has a relatively stronger Raman signal around 1431 ${\mathrm{cm}}^{-1}$ and a more intense amide III band with peaks at 1610 ${\mathrm{cm}}^{-1}$ and 1670 ${\mathrm{cm}}^{-1}$ the ASI-complex has a more pronounced amide I band with a maximum at 1300 ${\mathrm{cm}}^{-1}$ (Fig. 7(d)). Since the ASI-complex also contains the spike RBD, the peak of β -sheet structures around 1630 ${\mathrm{cm}}^{-1}$ can also be found here. This has the effect that Raman signals in the AS-complex between 1600 ${\mathrm{cm}}^{-1}$ and 1640 ${\mathrm{cm}}^{-1}$ are still more intense but the peak is shifted to 1610 ${\mathrm{cm}}^{-1}$ . However, the differences between the AS-complex and the ASI-complex are similar to the differences between the spike RBD and the ACE2 receptor. This can be investigated by the PCA of all four types of samples (Fig. 7(c)). Here, the measurements of the spike RBD and ACE2 can also be separated very well by the first principal component of this analysis. Overall, the AS-complex and the ASI-complex are more similar to the characteristics of the ACE2 receptor. While most of the measurements of the ASI-complex are overlapping with the measurements of ACE2, the AS-complex measurements are shifted towards negative PC1 values and therefore contain more features of the spike RBD. Since the features of the first PC in this comparison are very similar to the the comparison of the spike RBD to ACE2 (Fig. 7(d)), main differences between the spike RBD bound to ACE2 and the unbound state can be described by features of the spike RBD and ACE2. In conclusion the PCA also reveals that in case the spike RBD binds to the ACE2 receptor (AS-complex) more features of the spike RBD and of a β -sheet structure can be investigated in the Raman spectra. If the binding is prevented by the inhibitor cohaerin C (ASI-complex), features of the ACE2 receptor and of a α -helix structure are more dominant. The results also show that a distinction of the different samples and the bound and unbound state can be done very well with this method.

## 4. Discussion

The SERS-based approach demonstrate that differences and changes mainly in the region of the amide bands could be observed. Bound and unbound states of ACE2 and spike RBD can be distinguished by signals in the range of the α -helix and the β -sheet structure as well as by features of the SERS spectra of ACE2 and the spike RBD.

To understand the interpretation and results better, potential influences are discussed in the following. To protect the proteins from external influences, the samples have to be prepared accordingly. For the preparation of the SERS substrate an overnight incubation of the proteins in a wet chamber was chosen as such mild conditions ensure the optimal protection of the protein’s secondary structures. Also, for the comparison, all samples were prepared using the same preparation procedure as described in section 2.4. However, it is possible that influences from the SERS substrate or the PBS buffer occur.

Since SERS is a surface sensitive technique, in general signals of the whole structure within a protein complex might not be revealed. Also, Raman signals might be enhanced differently and influences of the SERS substrate are possible. As mentioned in section 3.1, the signals assigned to the ACE2 structure in mixed samples are dominant and more enhanced in the region 800 ${\mathrm{cm}}^{-1}$ to 1200 ${\mathrm{cm}}^{-1}$ . Also, the glass background of the SERS substrate overlaps with signals of ACE2 and of the mixed samples. Consequently, a analysis and comparison in this region is challenging. In the region 1200 ${\mathrm{cm}}^{-1}$ to 1750 ${\mathrm{cm}}^{-1}$ influences of the SERS substrate are smaller. Nevertheless, slightly different enhancements of Raman signals of protein structures are possible. From the deconvolution of the amide bands and comparison to the X-ray diffraction data it can be seen that in individual samples the determined distribution of α -helical and β -sheet structures is similar. In case of the AS-complex the distribution differs the most. Here, the spike RBD binds to ACE2 and features of the SERS spectrum of the spike RBD are more visible. Therefore, it is possible that additionally to a structural change the SERS effect enhance signals of the spike RBD structure more when this structure is coupled to the ACE2 structure. In that case the calculated distribution of the secondary structure might be a little different. However, since only the Raman spectra of the mixed samples contains significant more features of the spike RBD structure when the spike RBD binds to the ACE2 receptor, this characteristic can be used to differentiate between bound and unbound states. Regarding the ASI-complex, the determined structure from the SERS spectra is more similar to the one of the X-ray diffraction analysis and features of the spike RBD are less enhanced.

In addition to the possible influences from the SERS substrate, confounding factors caused by backgrounds such as fluorescence signals from the PBS buffer, structural changes or the inhibitor in the ASI-complex can also affect the results. In case of the fluorescence, the signal is very broad and minor influences on the analysis are possible (Fig. 3(d)). Since the aim of this work is not to determine exact distributions of the protein structures and all samples were prepared in the same PBS buffer, it cannot be expected that influences from fluorescence signals have an significant impact on the performed analysis. The SERS spectrum of the pure inhibitor reveals peaks around 1135 ${\mathrm{cm}}^{-1}$ , 1252 ${\mathrm{cm}}^{-1}$ and 1366 ${\mathrm{cm}}^{-1}$ and not in the range of the amide I band (Fig. 3(c)). These peaks could not be observed in the spectra of the ASI-complex. A structural change and therefore a change of the Raman signal of the inhibitor in the complex is also possible. However, since signals of the ACE2 structure seem to be enhanced more effectively in mixed samples on the SERS substrate, it can also be assumed that the influence of signals of the inhibitor itself is relatively weak in the complex.

Regarding the analysis methods, the deconvolution of the amide bands can be influenced by noise and results might have a larger uncertainty. Consequently, considering also possible previous discussed influences, this analysis method enables a good first insight into the structure and the determination whether a certain structure exist in more quantity or not is possible. Nevertheless, a determination of exact amounts of secondary structures is challenging. However, the multivariate data analysis, here performed with principal component analysis (PCA), is more consistent and features of the SERS spectra of each samples can be determined. In the PCA also the ratio of the signal strength of the amide bands is considered. The performed analysis shows that both analysis methods show similar results and in combination the Raman spectra can be investigated in more detail.

Regarding the whole measurement complex, consisting of the optical setup, the SERS substrate and the preparation, on the one side different factors need to be considered, but on the other side the analysis and the results show that an estimation of the secondary structure and a determination of bound and unbound states in the ACE2 and spike RBD complex is possible.

In future work further investigations of different inhibitors, other protein complexes and different SERS substrates are planned to understand possible influences better and enable a more precise structural analysis. Moreover, the measurement process can be optimised using better adapted SERS substrates or a improved optical system. Specifications of the SERS substrate used in this work can be seen in section 2.2. The deviation of the substrate is about 10% and a more consistent SERS substrate can lead to a more precise measurement. In this study, a concentration of 9 μ M of the proteins was chosen based on previous work by Jansen-Olliges et al. [26] to generate a sufficiently strong signal for the SERS analysis. With the optical setup the signal-to-noise ratio (SNR) of the amide bands varies between 25 to 60 and the SERS substrate provides a relative enhancement ratio up to ${\mathrm{10}}^{6}$ . As the amide bands exhibit a relatively broad Raman band, measurements at lower concentrations might be possible. However, since the focus of this study was not the sensitivity, in further studies also detection limits will be investigated.

## 5. Conclusion

In conclusion, we demonstrated that by using surface enhanced Raman spectroscopy a fast structural analysis of the ACE2 receptor, the spike RBD and the changes caused by their binding activities is possible. To prove this, samples of ACE2, the spike RBD and mixed samples where the spike RBD binds to ACE2 (AS-complex) and the binding is prevented by the inhibitor fungal cohaerin C (ASI-complex) were investigated with deconvolution of the amide bands and principal component analysis. Using deconvolution and PCA to analyse the SERS spectra of the four different scenarios, the relative amount of α -helical and β -sheet structures could be estimated and a distinction of ACE2, the spike RBD and its bound and unbound states was possible. In ACE2 and the ASI-complex stronger signals of α -helical structures were found while the SERS spectra of the spike RBD contain more characteristics of β -sheet structures. In case of the AS-complex, where the spike RBD binds to ACE2, more features of the spike RBD could be observed in the SERS spectra. In summary the SERS-based approach enables a rapid and label-free investigation of the spike RBD and the ACE2 structures as well as insights into the changes due to binding interactions. Furthermore, the approach can be used to observe the functionality of the inhibitor. Therefore, future sensing and screening applications not only for spike RBD and ACE2 but also for protein binding investigations in general appear feasible.

## Data Availability

Data underlying the results presented in this paper can be obtained from the authors upon reasonable request.

## References

[1] Hu B. Guo H. Zhou P. Shi Z.-L., “Characteristics of SARS-CoV-2 and COVID-19,” Nat. Rev. Microbiol. 19(3), 141–154 (2021). 10.1038/s41579-020-00459-7 33024307 PMC7537588

[2] Telenti A. Hodcroft E. B. Robertson D. L., “The evolution and biology of SARS-CoV-2 variants,” Cold Spring Harbor Perspect. Med. 12(5), a041390 (2022). 10.1101/cshperspect.a041390 PMC9159258 35444005

[3] Koelle K. Martin M. A. Antia R. Lopman B. Dean N. E., “The changing epidemiology of SARS-CoV-2,” Science 375(6585), 1116–1121 (2022). 10.1126/science.abm4915 35271324 PMC9009722

[4] Otto S. P. Day T. Arino J. Colijn C. Dushoff J. Li M. Mechai S. Van Domselaar G. Wu J. Earn D. J. Odgen N. H., “The origins and potential future of SARS-CoV-2 variants of concern in the evolving COVID-19 pandemic,” Curr. Biol. 31(14), R918–R929 (2021). 10.1016/j.cub.2021.06.049 34314723 PMC8220957

[5] Maher M. C. Bartha I. Weaver S. Di Iulio J. Ferri E. Soriaga L. Lempp F. A. Hie B. L. Bryson B. Berger B. Robertson D. L. Snell G. Corti D. Virgin H. W. Kosakovsky Pond S. L. Telenti A., “Predicting the mutational drivers of future SARS-CoV-2 variants of concern,” Sci. Transl. Med. 14(633), eabk3445 (2022). 10.1126/scitranslmed.abk3445 35014856 PMC8939770

[6] Lan J. Ge J. Yu J. Shan S. Zhou H. Fan S. Zhang Q. Shi X. Wang Q. Zhang L. Wang X., “Structure of the SARS-CoV-2 spike receptor-binding domain bound to the ACE2 receptor,” Nature 581(7807), 215–220 (2020). 10.1038/s41586-020-2180-5 32225176

[7] Song W. Gui M. Wang X. Xiang Y., “Cryo-EM structure of the SARS coronavirus spike glycoprotein in complex with its host cell receptor ACE2,” PLoS Pathog. 14(8), e1007236 (2018). 10.1371/journal.ppat.1007236 30102747 PMC6107290

[8] Dinnes J. Sharma P. Berhane S. et al., “Rapid, point-of-care antigen tests for diagnosis of SARS-CoV-2 infection,” Cochrane Database Syst. Rev. 2022(7), CD013705 (2022). 10.1002/14651858.CD013705.pub3 PMC9305720 35866452

[9] Pezzotti G. Boschetto F. Ohgitani E. Fujita Y. Shin-Ya M. Adachi T. Yamamoto T. Kanamura N. Marin E. Zhu W. Nishimura I. Mazda O., “Raman molecular fingerprints of SARS-CoV-2 British variant and the concept of Raman barcode,” Adv. Sci. 9(3), 2103287 (2022). 10.1002/advs.202103287 PMC8787433 34877818

[10] Ventura B. D. Cennamo M. Minopoli A. Campanile R. Censi S. B. Terracciano D. Portella G. Velotta R., “Colorimetric test for fast detection of SARS-CoV-2 in nasal and throat swabs,” ACS Sens. 5(10), 3043–3048 (2020). 10.1021/acssensors.0c01742 32989986 PMC7534800

[11] Seo G. Lee G. Kim M. J. Baek S.-H. Choi M. Ku K. B. Lee C.-S. Jun S. Park D. Kim H. G. Kim S.-J. Lee J.-O. Kim B. T. Park E. C. Kim S. I., “Rapid detection of COVID-19 causative virus (SARS-CoV-2) in human nasopharyngeal swab specimens using field-effect transistor-based biosensor,” ACS Nano 14(4), 5135–5142 (2020). 10.1021/acsnano.0c02823 32293168

[12] López-Valls M. Escalona-Noguero C. Rodríguez-Díaz C. Pardo D. Castellanos M. Milán-Rois P. Martínez-Garay C. Coloma R. Abreu M. Cantón R. Galán J. C. Miranda R. Somoza Á. Sot B., “CASCADE: Naked eye-detection of SARS-CoV-2 using Cas13a and gold nanoparticles,” Anal. Chim. Acta 1205, 339749 (2022). 10.1016/j.aca.2022.339749 35414398 PMC8939626

[13] Yan R. Zhang Y. Li Y. Xia L. Guo Y. Zhou Q., “Structural basis for the recognition of SARS-CoV-2 by full-length human ACE2,” Science 367(6485), 1444–1448 (2020). 10.1126/science.abb2762 32132184 PMC7164635

[14] Han P. Li L. Liu S. et al., “Receptor binding and complex structures of human ACE2 to spike RBD from omicron and delta SARS-CoV-2,” Cell 185(4), 630–640.e10 (2022). 10.1016/j.cell.2022.01.001 35093192 PMC8733278

[15] Kim K. Kashefi-Kheyrabadi L. Joung Y. Kim K. Dang H. Chavan S. G. Lee M.-H. Choo J., “Recent advances in sensitive surface-enhanced Raman scattering-based lateral flow assay platforms for point-of-care diagnostics of infectious diseases,” Sensors and Actuators B: Chemical 329, 129214 (2021). 10.1016/j.snb.2020.129214 36568647 PMC9759493

[16] Kniggendorf A.-K. Meinhardt-Wollweber M. Yuan X. Roth B. Seifert A. Fertig N. Zeilinger C., “Temperature-sensitive gating of hCx26: high-resolution Raman spectroscopy sheds light on conformational changes,” Biomed. Opt. Express 5(7), 2054–2065 (2014). 10.1364/BOE.5.002054 25071948 PMC4102348

[17] Kniggendorf A.-K. Schmidt D. Roth B. Plettenburg O. Zeilinger C., “pH-Dependent Conformational Changes of KcsA Tetramer and Monomer Probed by Raman Spectroscopy,” Int. J. Mol. Sci. 20(11), 2736 (2019). 10.3390/ijms20112736 31167355 PMC6601014

[18] Kniggendorf A.-K. Nogueira R. Nasiri Bahmanabad S. Pommerening-Röser A. Roth B. W., “Small sample stress: Probing oxygen-deprived ammonia-oxidizing bacteria with Raman spectroscopy in vivo,” Microorganisms 8(3), 432 (2020). 10.3390/microorganisms8030432 32204374 PMC7143505

[19] Kniggendorf A.-K. Nogueira R. Lorey C. Roth B., “Calcium carbonate deposits and microbial assemblages on microplastics in oligotrophic freshwaters,” Chemosphere 266, 128942 (2021). 10.1016/j.chemosphere.2020.128942 33220990

[20] Yue W. Xia Z. Zeng Z. Chen Z. Qiao L. Li P. He Y. Luo X., “In situ surface-enhanced Raman scattering detection of a SARS-CoV-2 biomarker using flexible and transparent polydimethylsiloxane films with embedded Au nanoplates,” ACS Appl. Nano Mater. 5(9), 12897–12906 (2022). 10.1021/acsanm.2c02750 37552747

[21] Abdullah M. B. Dab C. Almalki M. Alnaim A. Abuzir A. Awada C., “Ultrafast Detection of SARS-CoV-2 Spike protein (S) and receptor-binding domain (RBD) in Saliva using surface-enhanced Raman spectroscopy,” Appl. Sci. 12(10), 5039 (2022). 10.3390/app12105039

[22] Sanchez J. E. Jaramillo S. A. Settles E. Salazar J. J. V. Lehr A. Gonzalez J. Aranda C. R. Navarro-Contreras H. R. Raniere M. O. Harvey M. Wagner D. M. Koppisch A. Kellar R. Keim P. Yacaman M. J., “Detection of SARS-CoV-2 and its S and N proteins using surface enhanced Raman spectroscopy,” RSC Adv. 11(41), 25788–25794 (2021). 10.1039/D1RA03481B 35478863 PMC9037103

[23] Yang Y. Peng Y. Lin C. Long L. Hu J. He J. Zeng H. Huang Z. Li Z.-Y. Tanemura M. Shi J. Lombardi J. R. Luo X., “Human ACE2-functionalized gold virus-trap nanostructures for accurate capture of SARS-CoV-2 and single-virus SERS detection,” Nano-Micro Lett. 13(1), 109–113 (2021). 10.1007/s40820-021-00620-8 PMC8042470 33868761

[24] Leong S. X. Leong Y. X. Tan E. X. et al., “Noninvasive and point-of-care surface-enhanced Raman scattering (SERS)-based breathalyzer for mass screening of coronavirus disease 2019 (COVID-19) under 5 min,” ACS Nano 16(2), 2629–2639 (2022). 10.1021/acsnano.1c09371 35040314

[25] Huang J. Wen J. Zhou M. et al., “On-site detection of SARS-CoV-2 antigen by deep learning-based surface-enhanced Raman spectroscopy and its biochemical foundations,” Anal. Chem. 93(26), 9174–9182 (2021). 10.1021/acs.analchem.1c01061 34155883

[26] Jansen-Olliges L. Chatterjee S. Jia L. Stahl F. Bär C. Stadler M. Surup F. Zeilinger C., “Multiformin-Type Azaphilones Prevent SARS-CoV-2 Binding to ACE2 Receptor,” Cells 12(1), 83 (2022). 10.3390/cells12010083 36611877 PMC9818685

[27] Bremer K. Roth B., “Fibre optic surface plasmon resonance sensor system designed for smartphones,” Opt. Express 23(13), 17179–17184 (2015). 10.1364/OE.23.017179 26191726

[28] Walter J.-G. Eilers A. Alwis L. S. M. Roth B. W. Bremer K., “SPR biosensor based on polymer multi-mode optical waveguide and nanoparticle signal enhancement,” Sensors 20(10), 2889 (2020). 10.3390/s20102889 32443702 PMC7287642

[29] Walter J.-G. Alwis L. S. Roth B. Bremer K., “All-optical planar polymer waveguide-based biosensor chip designed for smartphone-assisted detection of vitamin D,” Sensors 20(23), 6771 (2020). 10.3390/s20236771 33260818 PMC7730180

[30] Surup F. Mohr K. I. Jansen R. Stadler M., “Cohaerins G–K, azaphilone pigments from Annulohypoxylon cohaerens and absolute stereochemistry of cohaerins C–K,” Phytochemistry 95, 252–258 (2013). 10.1016/j.phytochem.2013.07.027 23969107

[31] Kniggendorf A. K. Wetzel C. Roth B., “Microplastics detection in streaming tap water with Raman spectroscopy,” Sensors 19(8), 1839 (2019). 10.3390/s19081839 31003396 PMC6515371

[32] Koch M. Suhr C. Roth B. Meinhardt-Wollweber M., “Iterative morphological and mollifier-based baseline correction for Raman spectra,” J. Raman Spectrosc. 48(2), 336–342 (2017). 10.1002/jrs.5010

[33] Sadat A. Joye I. J., “Peak fitting applied to fourier transform infrared and Raman spectroscopic analysis of proteins,” Appl. Sci. 10(17), 5918 (2020). 10.3390/app10175918

[34] Baldassarre M. Li C. Eremina N. Goormaghtigh E. Barth A., “Simultaneous fitting of absorption spectra and their second derivatives for an improved analysis of protein infrared spectra,” Molecules 20(7), 12599–12622 (2015). 10.3390/molecules200712599 26184143 PMC6331840

[35] Maiti N. C. Apetri M. M. Zagorski M. G. Carey P. R. Anderson V. E., “Raman spectroscopic characterization of secondary structure in natively unfolded proteins: *α*-synuclein,” J. Am. Chem. Soc. 126(8), 2399–2408 (2004). 10.1021/ja0356176 14982446

[36] Nemecek D. Stepanek J. Thomas Jr G. J., “Raman spectroscopy of proteins and nucleoproteins,” Curr. protocols in protein science 71(1), 17–18 (2013). 10.1002/0471140864.ps1708s71 23377849

[37] Rygula A. Majzner K. Marzec K. M. Kaczor A. Pilarczyk M. Baranska M., “Raman spectroscopy of proteins: a review,” J. Raman Spectrosc. 44(8), 1061–1076 (2013). 10.1002/jrs.4335

[38] Kuhar N. Sil S. Verma T. Umapathy S., “Challenges in application of Raman spectroscopy to biology and materials,” RSC Adv. 8(46), 25888–25908 (2018). 10.1039/C8RA04491K 35541973 PMC9083091

[39] Tuma R., “Raman spectroscopy of proteins: from peptides to large assemblies,” J. Raman Spectrosc. 36(4), 307–319 (2005). 10.1002/jrs.1323

[40] Chi Z. Chen X. Holtz J. S. Asher S. A., “UV resonance Raman-selective amide vibrational enhancement: quantitative methodology for determining protein secondary structure,” Biochemistry 37(9), 2854–2864 (1998). 10.1021/bi971160z 9485436

[41] Ngarize S. Herman H. Adams A. Howell N., “Comparison of changes in the secondary structure of unheated, heated, and high-pressure-treated *β*-lactoglobulin and ovalbumin proteins using Fourier transform Raman spectroscopy and self-deconvolution,” J. Agric. Food Chem. 52(21), 6470–6477 (2004). 10.1021/jf030649y 15479009

[42] Shang J. Ye G. Shi K. Wan Y. Luo C. Aihara H. Geng Q. Auerbach A. Li F., “Structural basis of receptor recognition by SARS-CoV-2,” Nature 581(7807), 221–224 (2020). 10.1038/s41586-020-2179-y 32225175 PMC7328981

[43] Berman H. M. Westbrook J. Feng Z. Gilliland G. Bhat T. N. Weissig H. Shindyalov I. N. Bourne P. E., “The protein data bank,” Nucleic Acids Res. 28(1), 235–242 (2000). 10.1093/nar/28.1.235 10592235 PMC102472

[44] Kabsch W. Sander C., “Dictionary of protein secondary structure: pattern recognition of hydrogen-bonded and geometrical features,” Biopolym. Orig. Res. on Biomol. 22(12), 2577–2637 (1983). 10.1002/bip.360221211 6667333

[45] Klose D. Wallace B. A. Janes R. W., “2Struc: the secondary structure server,” Bioinformatics 26(20), 2624–2625 (2010). 10.1093/bioinformatics/btq480 20739308 PMC2951091

